# Solar Radiation and Tidal Exposure as Environmental Drivers of *Enhalus acoroides* Dominated Seagrass Meadows

**DOI:** 10.1371/journal.pone.0034133

**Published:** 2012-03-29

**Authors:** Richard K. F. Unsworth, Michael A. Rasheed, Kathryn M. Chartrand, Anthony J. Roelofs

**Affiliations:** 1 Centre for Sustainable Aquatic Research, College of Science, Swansea University, Swansea, Wales, United Kingdom; 2 Northern Fisheries Centre, Fisheries Queensland, Cairns, Australia; University of Western Australia, Australia

## Abstract

There is strong evidence of a global long-term decline in seagrass meadows that is widely attributed to anthropogenic activity. Yet in many regions, attributing these changes to actual activities is difficult, as there exists limited understanding of the natural processes that can influence these valuable ecosystem service providers. Being able to separate natural from anthropogenic causes of seagrass change is important for developing strategies that effectively mitigate and manage anthropogenic impacts on seagrass, and promote coastal ecosystems resilient to future environmental change. The present study investigated the influence of environmental and climate related factors on seagrass biomass in a large ≈250 ha meadow in tropical north east Australia. Annual monitoring of the intertidal *Enhalus acoroides* (L.f.) Royle seagrass meadow over eleven years revealed a declining trend in above-ground biomass (54% significant overall reduction from 2000 to 2010). Partial Least Squares Regression found this reduction to be significantly and negatively correlated with tidal exposure, and significantly and negatively correlated with the amount of solar radiation. This study documents how natural long-term tidal variability can influence long-term seagrass dynamics. Exposure to desiccation, high UV, and daytime temperature regimes are discussed as the likely mechanisms for the action of these factors in causing this decline. The results emphasise the importance of understanding and assessing natural environmentally-driven change when interpreting the results of seagrass monitoring programs.

## Introduction

There is strong evidence of a global long-term decline in seagrass meadows related to anthropogenic activity [1]. Specific causes of this decline have been linked to a range of factors including reduced water quality, dredging, and coastal and port development [2], [3], [4]. While it is accepted that anthropogenic activities can affect seagrass health, seagrasses are also impacted by a range of natural drivers, including variability in climate and hydrodynamic conditions, both seasonally and among years [5], [6], [7]. Separating natural from anthropogenic causes of seagrass change is important for developing strategies that effectively mitigate and manage anthropogenic impacts on seagrass and promote coastal ecosystems resilient to future environmental change.

Intertidal seagrass meadows form an ecologically and economically important component of coastal ecosystems [8]. In places where turbidity is naturally high, seagrasses are often restricted exclusively to the intertidal zone [9]. These intertidal seagrasses are particularly vulnerable to changes in light levels, temperature and the duration of emersion and exposure [3], [10], [11]. Such factors all have the potential to be influenced by the interacting effects of climate change, localised pollution and a degraded ozone layer, resulting in conditions potentially damaging to seagrass [12], [13].

During tidal exposure, intertidal seagrasses are susceptible to extreme irradiance doses, desiccation [14], thermal stress [10] and potentially high UV-A and UV-B [15], [16] leading to physiological damage. However, the periods around and even during exposure may provide critical windows of sufficient light for positive net photosynthesis [17].

Low light adapted Angiosperms subjected to high light commonly exhibit a stress response which may include photoinhibition, altered photosynthetic pigments, and morphological changes [18]. Elevated UV-A, and UV-B have also been shown to cause a decline in photosynthetic efficiency [15], with some species having a higher capacity than others to conduct photo repair after light related photosynthetic inhibition [16].

The effects of high light and temperature coupled to periods of tidal exposure have been documented to result in short-term declines in seagrass density and spatial coverage [19], [20], [21]. For example, a combination of summer time tidal exposure, high light and high temperatures are thought to have caused the loss of 13000 hectares (ha) of seagrass meadow in South Australia [21]. However, no studies have considered how tidal exposure over multiple years may influence seagrass dynamics, especially when interacting factors such as variability of temperature and solar radiation are considered. Long-term lunar cycles affect the number of daytime hours an intertidal flat is exposed to the air and this relationship changes from year to year.

Long-term seagrass dynamics of many tropical seagrasses are poorly understood, particularly within the Indo-Pacific region. Specifically, the temporal dynamics of the species *Enhalus acoroides* and its response to environmental factors have received little attention over the long-term. These issues have enormous importance for the extensive large closed canopy habitat that *E. acoroides* provides for diverse, endangered and economically important fauna throughout the Indo-Pacific bio-region [22], [23].

Intertidal *E. acoroides* meadows are found throughout the Indo-Pacific region and the north-eastern region of Australia. In many instances, *E. acoroides* meadows are adjacent to ports, shipping lanes and large coastal developments [24]. Such localities are of high environmental risk, largely due to potential impacts from regular dredging and infrastructure development [3].

The objective of this study was to examine the long-term temporal dynamics of intertidal *E. acoroides* seagrass in the Northern region of the Gulf of Carpentaria (NE Australia) to determine whether environmental factors related to tidal exposure cycles correlate to changes in seagrass above-ground biomass and if so, at what temporal scale these factors most influence the seagrass.

## Materials and Methods

**Figure 1:**
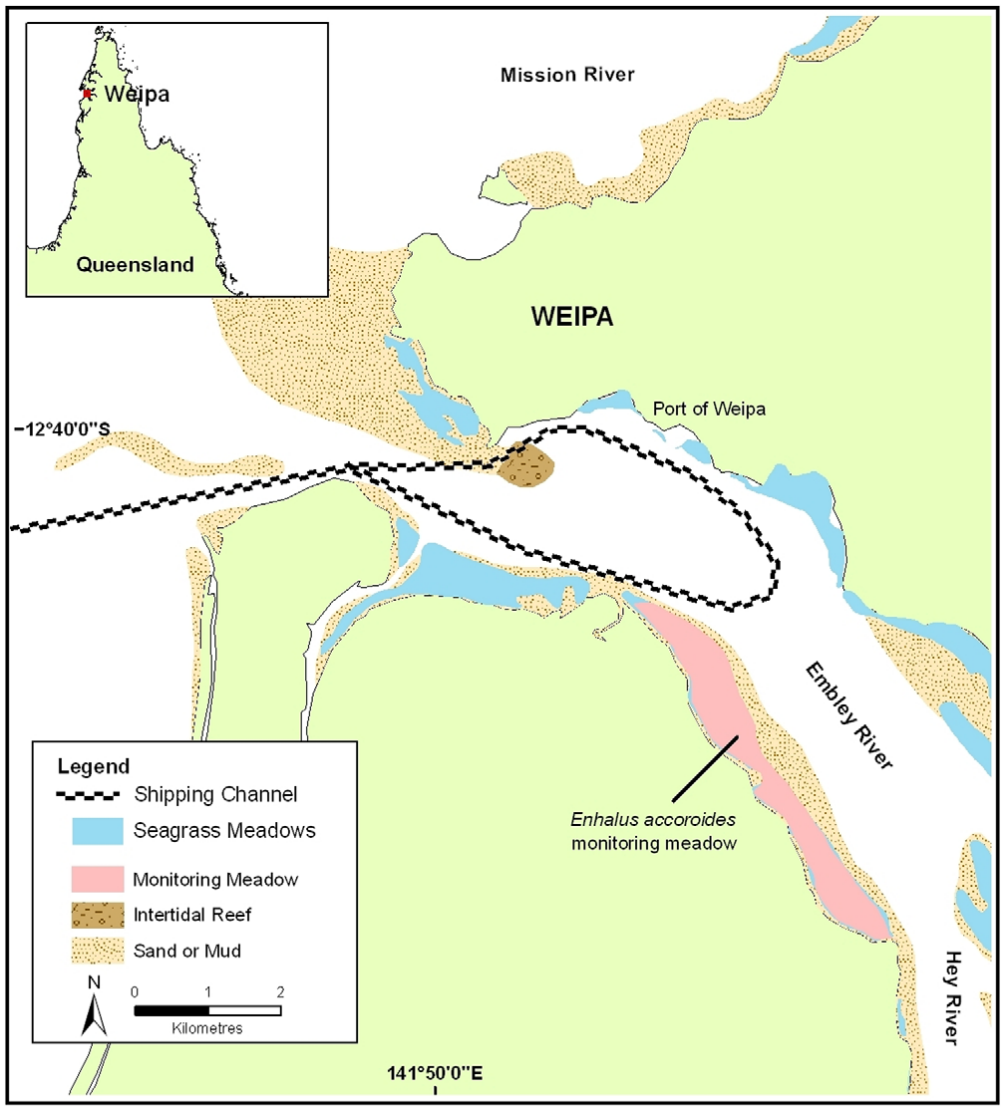
Location of the seagrass meadow near the town of Weipa, Queensland, Australia.

Seagrass distribution and above-ground biomass were measured within an intertidal seagrass meadow (≈250 ha) on the shallow mud and sand banks of the mouth of the Embley River adjacent to Weipa, Queensland, North East Australia (Figure 1). The meadow was dominated by the large bladed seagrass species *Enhalus acoroides* (L.F.) Royle. A very low density (<5% of total biomass) of other smaller species including *Thalassia hemprichii* (Ehrenberg) Ascherson, *Halodule uninervis* Forsskål (Ascherson), and *Halophila ovalis* R. Brown were interspersed. The meadow is adjacent to a bulk export port (bauxite) and associated shipping channel. Volume of material removed annually, and the duration of the annual dredging campaign ranged between 0 and 3 million m^3^ and 0 and 99 days per year respectively, with no apparent trends in volume or duration over the eleven year period. The seagrass meadow was monitored annually to assess its condition in relation to port activities including annual dredging of the shipping lane. Previous analysis (that includes the use of independent reference sites) has found no significant correlation between dredging and the health and productivity of the meadow despite declines in meadow biomass without explanation [25].

Annual surveys from 2000 to 2010 were conducted during the late dry season (August–September) when N.E Australian seagrass abundance is typically at its seasonal peak [5]. The seagrass monitoring program in Weipa followed the defined methodology used in other seagrass research programs throughout Queensland [7], [24]. Each year, the seagrass meadow boundary of the intertidal *E. acoroides* dominated meadow was mapped by aerial (helicopter) survey. This was done when the meadow was exposed at low tide and involved the use of a global positioning system (GPS) and a Geographic Information System (GIS) basemap [26]. The precision of determining seagrass meadow boundaries was expressed as an estimate of reliability (R) [26]. Seagrass habitat characteristics (seagrass species composition and above-ground biomass) were described at sites scattered randomly each year within the seagrass meadow (sites were therefore fully independent with respect to year). A power analysis based on the initial survey conducted in 2000 determined the number of sites placed within the meadow [27]. As the meadow changed in biomass the power analysis was revised to maintain statistical power. GPS fixes were recorded at each sampling site from a helicopter hovering within one metre of the ground. A visual estimate of biomass technique [28], [29] was used to estimate above-ground biomass at each site [7], [24].

### Climate Data

Data on water characteristics (e.g. temperature, light availability) was unavailable for Weipa, therefore an analysis of the environmental influences on seagrass focused upon the effect of four main factors: air temperature, rainfall, solar radiation (global solar radiation) and daytime tidal exposure. All climate and tidal data used within this study are publicly available from the Australian Bureau of Meteorology [30] and Maritime Safety Queensland (provided on behalf of the Coastal Sciences Unit Environmental Sciences Division of the Environment Protection Agency (EPA)). Climate data were collected from the nearest weather station at Weipa Airport (station #027045).

Global solar exposure is the total amount of solar energy falling on a horizontal surface [30]. Typical values for total daily global solar exposure range from 1 to 35 MJ/m^2^ (megajoules per square metre) [30].

For each of the eleven years, a mean value for all environmental factors for the previous 1, 3, 6, 9 and 12 months prior to seagrass monitoring was determined. This created five separate variables for each individual climate factor. These 5 different durations represent biologically meaningful changes in environmental conditions within these meadows due to lunar (1 month), seasonal (3, 6 and 9 months) and annual cycles (12 months). Although tropical seasonal variability in north east Australia is typically only described as wet or dry, these two seasons can be further split in into the dry, late dry, monsoon, and late monsoon. This is because the variability in temperature, wind and rainfall associated with these periods [5]. Previous analysis of environmental factors influencing seagrass meadows in NE Australia have revealed similar periods of duration to be influential [7].

An index of tidal exposure was also created for the Weipa meadow. The total monthly daylight hours that the tidal height was recorded to be less than 1.0 m was calculated over the eleven year period (i.e. the point at which the meadow became exposed). The total daylight hours for the 1, 3, 6, 9 and 12 months periods were then determined for each year. In total, the 3 environmental factors and the exposure index therefore created 20 separate variables for use in regression analysis (see Table 1).

**Table 1:** Climate and environmental factors together with their averaging times used in Partial Least Squares (PLS) regression analysis of seagrass meadow changes at Weipa, North Queensland, Australia (2000 to 2010).

Factor	Averaging time used in PLS regression
Maximum daily air temperature (°C)	Previous 1, 3, 6, 9 and 12 months
Total monthly rainfall (mm)	Previous 1, 3, 6, 9 and 12 months
Total daily solar radiation (MJ/m^2^)	Previous 1, 3, 6, 9 and 12 months
Total monthly daylight tidal exposure (hours)	Previous 1, 3, 6, 9 and 12 months

### Data analysis

Summary statistics of seagrass data were calculated and all mean values are displayed together with their standard errors. All observations taken each year were randomly distributed and therefore considered completely independent observations allowing inter-annual comparisons and regression analysis. Data was not normally distributed and differences in mean biomass between years were therefore analysed using one-way Kruskal-Wallis on ranks within SigmaPlot v11.

To investigate which of the twenty variables correlated most with annual mean seagrass meadow biomass and area within each meadow, a Partial Least Squares Regression (PLS) model was developed in Minitab (version 16) [7], [31]. PLS regression is particularly suited to incidences when the matrix of predictors has more variables than observations, and when there is multi co-linearity among variables [32]. The study had eleven observations (years) and twenty variables for the meadow, and many of the variables were co-linear. This technique has commonly been used to analyse a range of ecological datasets [32]. Due to measurements being taken over time any concerns with respect to potential auto-correlation were considered negligible after calculating the Durbin–Watson statistic [33].

Annual mean total seagrass biomass data for the whole species assemblage and the annual total meadow area were analysed against the twenty variables. PLS was conducted in a step-wise manner that allowed for the successive removal of variables that did not contribute to the model, enabling the strongest possible PLS model to be created. The PLS analysis also calculated a predicted residual sum of squares (PRESS) following cross-validation.

## Results

**Figure 2:**
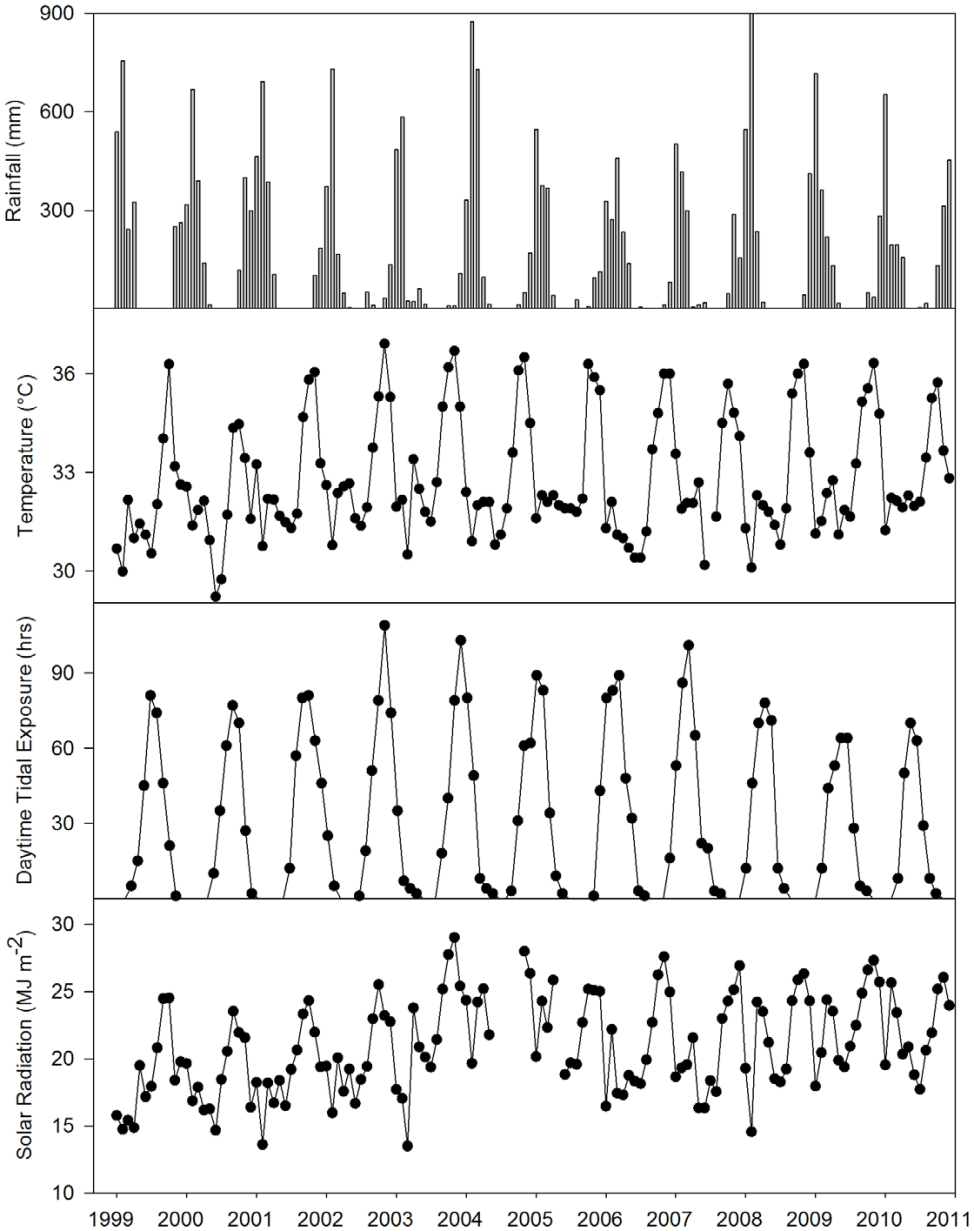
Monthly environmental parameters between 2000 and 2010 at Weipa Airport, Far North Queensland, Australia. (a) Total monthly rainfall, (b) Mean daily maximum air temperature (Temp), (c) Total daytime hours that the seagrass meadow was subjected to a tidal height <1.0 m (d) Mean daily global solar exposure.

Environmental conditions in Weipa were highly variable both within and among years from 1999 to 2010 (see Figure 2). Seasonality was pronounced, with temperature reaching a maximum during the austral summer (December and January) and a minimum during winter (June and July). Rainfall also varied seasonally with maximum rainfall observed during February and March (Figure 2).

Maximum average daily temperature was highly variable among years, with an annual monthly mean of 32.8±0.1°C, this was lowest in 2000 with a mean of 31.9°C and at a maximum of 33.3°C in 2003. Rainfall was at its minimum in 2003 with a total of 1320 mm and at a maximum in 1999 with a total of 2375 mm of rain (Figure 2). Average annual rainfall from 1999 to 2009 was 1879.4±111.6 mm. Annual Solar radiation averaged 21.0±0.5 MJ m^−2^ from 1999 to 2010, reaching a max of 24.2 in 2004 and a minimum of 18.6 in 1999. Total hours of daytime tidal exposure also varied on an inter- and intra- annual basis. Generally, there was no daytime tidal exposure during December, January and February, which were the hottest months of the year. Daytime exposure followed a strong ‘seasonal type’ pattern with an annual peak reached regularly in July. Variability among years was also present. Daytime exposure was highest in 2004 at 383 hrs, and lowest in 2009 with 273 hrs. The annual mean total daytime tidal exposure was 339.1±15.2 hrs across all years.

**Figure 3:**
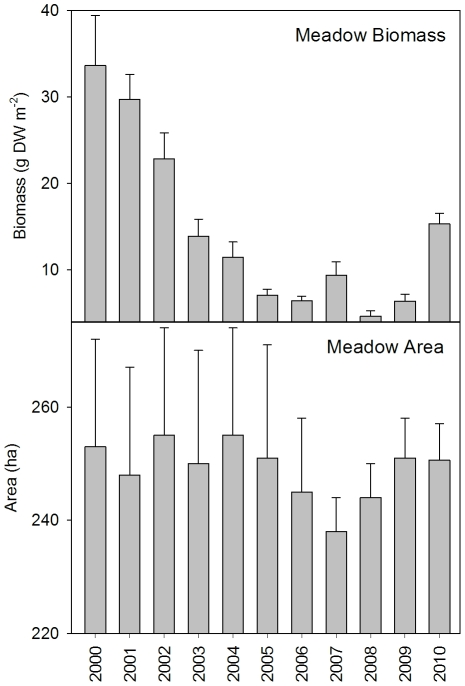
Mean (±SE) annual habitat parameters. (a) biomass and (b) area, recorded each August–September between 2000 and 2010 for an intertidal *Enhalus acoroides* seagrass meadow in Weipa, Far North Queensland, Australia.

During the eleven year period of observation, meadow biomass was highly variable while meadow area varied very little (Figure 3). Mean (±SE) meadow area was 249±1.7 ha, reaching a minimum of 238±6 ha in 2007 and a maximum of 255±19 ha in 2002. Mean meadow biomass over the eleven years was 14.6±3.0 gDW.m^−2^ with significant reductions of 54% (H_1,10_ = 118.692, P<0.001) from 2000 to 2010. Seagrass biomass was significantly and negatively correlated with tidal exposure during the previous month of observations, and significantly and negatively correlated with the amount of solar radiation during the previous 12 months of observations (Table 2).

**Table 2:** Partial least squares (PLS) regression analysis (final models following stepwise analysis) of annual mean seagrass biomass relative to available climate and environmental data at Weipa, North Queensland, Australia (2000 to 2010).

	PLS ANOVA			Model selection and validation				Predictors	
	P	F	DoF	Component	X Variance	R-Sq	R-Sq (pred)	Tidal Exp Previous Month	Solar Radiation previous 12 Months
ANOVA	<0.01	13.7	1,10						
PLS Model				1	0.55	0.60	0.21		
				2		0.61	0.19		
Coefficients								−0.42	−0.61

Table shows the overall ‘global’ ANOVA statistics for each of the regression models, the individual principal components and their cumulative R^2^ values. Individual regression coefficients of the specific biomass predictors (environmental variables) are also shown.

61% of the meadow biomass variability was explained by tidal exposure and solar radiation. After PLS cross validation (i.e. randomly removing 3 data points at a time then re-running the PLS analysis) this correlation remained significant and explained variability reduced to 19% (pred R-Sq), statistically indicating that the relationship was not driven exclusively by only 1 or 2 data points. There was no significant correlation between seagrass biomass and temperature or rainfall during the study period.

## Discussion

High light has long been documented to have short term negative impacts upon a range of marine flora [18], [34], [35] and several studies have shown the negative effects of thermal stress and desiccation on seagrasses [14], [20], [36]. The present study provides evidence that the long term variability in the quantity of solar energy, together with natural variations in daylight tidal exposure may have negative consequences on intertidal seagrass meadows. Given the predictable nature of daytime tidal exposure, these findings are of importance due to the increasing levels of stress from other less predictable environmental variables linked to climate change. As both seawater and terrestrial air temperatures increase with climate change [37] combined with increased exposure to elevated UV levels [38], [39], intertidal seagrass meadows are likely to experience far more extreme conditions and potentially be in a state less able to recover from natural environmental variability.

Intertidal *Enhalus acoroides* seagrass meadows represent a major ecological resource of value to a range of different fauna [40], [41], [42]. In the present study, above ground biomass over an eleven year period has varied widely, with biomass in 2009 at its lowest recorded level (<1/5^th^ its baseline value). Data is unavailable on the impact this large scale reduction in available habitat had on local fauna, but studies from elsewhere suggest there would likely be a significant negative impact. For example, large scale loss of seagrass (16%) in South Australia may have caused a 40% decline in the catch in the King George Whiting fishery [43].

Long-term analysis of change in such Indo-Pacific seagrass meadows is limited within the literature, particularly within meadows of *E. acoroides*. Here we report strong correlative evidence that long-term tidal cycles coinciding with daylight and high solar radiation are linked to this long-term variability and seagrass decline. This is in contrast to many studies that have found anthropogenic stress (i.e. localised pollution) to be causing large spatial scale patterns of seagrass decline [1]. Although there is irrefutable evidence of the impact of anthropogenic stress on seagrass, it is unlikely that all studies documenting seagrass decline completely exclude the impact of natural environmental variability upon those meadows.

A successful ecological monitoring programme needs to be able to explain the reasons for change when it does occur. This is critical when the ecological resource is within the range of impact of urban, industrial and agricultural activities. In Weipa there has been no evidence to suggest that temporal habitat variability was the result of anthropogenic impact [25], rather the present study used a wide range of environmental climate and tidal exposure data to demonstrate the natural drivers shifting meadow dynamics over time. This highlights the value of conducting long-term monitoring of seagrass meadows to understand patterns of natural variability versus those from anthropogenic change [24].

Other studies of Indo-Pacific seagrass meadows have found that long and frequent periods of tidal exposure can result in desiccation, temperature and high light stress, leading to permanent morphological and physiological damage to intertidal seagrasses [19], [20]. The mechanisms by which high solar radiation and exposure leads to seagrass decline are likely related to a combination of these factors causing physiological stress to the leaf structure and photosystems [14], [44]. The means by which solar radiation may act to cause increased physiological stress are probably through excess light causing photodamage. This occurs when excess irradiance causes the production of oxygen-free radicals, which in turn “damage” the photosynthetic apparatus [45].

Although our analysis of temperature excluded this as a direct correlate with seagrass biomass we used maximum daily air temperature and not actual *in situ* meadow temperature. In other studies of tropical seagrass meadows water remaining in shallow pools over seagrasses can become “super-heated” compared to surrounding water and air temperatures to the point where physiological damage to the seagrass plant occurred [10]. It is quite possible similar impacts could occur in Weipa so temperature cannot be excluded as contributing to seagrass exposure related stress.

**Figure 4:**
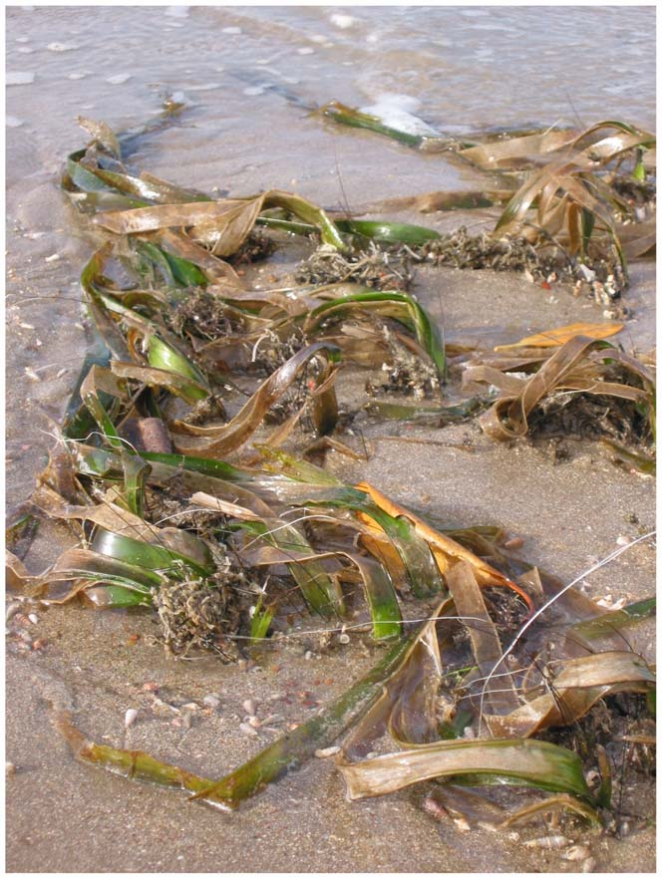
Tidal exposure and ‘burning’ of *Enhalus acoroides* at low tide in Weipa, Far North Queensland, Australia.

A study on seagrass resilience to desiccation has found that *E. acoroides* has a relatively high resistance due to the thick waxy leaves preventing water loss [14]. However, these morphological features may in reality make them more vulnerable to exposure-related loss than other intertidal species. The thick strap-like blades cause a portion of the blade base to remain “proud” above the substrate rather than lying flat on the surface (Figure 4).

Other species that lie flat on the surface when exposed may be more protected from the extremes of light, temperature and desiccation related stress [44], [46]. Intertidal meadows made up of small species with flexible petioles such as *Halodule uninervis* and *Halophila ovalis* in Weipa and elsewhere in north east Australia did not appear to suffer similar declines despite similar tidal regimes and exposure times [7].

Due to the correlative nature of the study it remains unclear as to whether light levels, temperature, or water loss is the critical physiological factor leading to loss in exposed intertidal *E. acoroides* meadows and to what degree morphological adaptations versus physiological tolerance determine resilience or susceptibility to exposure. Determining the direct cause would require further investigation to quantify their contributing effects.

Although the potential duration of tidal exposure varies between months, our analysis indicates that it is only the previous month that is important in influencing seagrass biomass. This indicates that the action of physiological stress driven by exposure is from a short period of stress, probably associated to desiccation and extreme heat, rather than as a persistent chronic stressor. The correlation between seagrass biomass and solar radiation however is over a 12 month period. This may be an indication that this stressor operates over longer periods to reduce the resilience of the meadow or perhaps that the critical impacts associated with solar irradiation occur at a different time in the year. High light and UV can cause a range of physiological stresses that can negatively impact the carbon balance of the plant through reduced photosynthesis and the need to expend increased energies on photo-repair [18], [34]. Such stress may act to reduce the resilience of the plant to further stressors during the year.

Due to the relatively quick shift away from tidal exposure stress, seagrass lost due to emersion and ‘burning’ in other locations has quickly recovered [19], preventing the potential shift of the habitat to an alternative stable state [47]. The persistent area of seagrass recorded throughout the eleven year study in Weipa is probably a reflection of the resilience of the meadow to periodic exposure related loss of the above ground structures. Typically, tropical seagrass species have a capacity for rapid recovery from loss particularly through asexual colonisation [48], [49]. Below ground carbohydrate reserves which were not examined in this study may provide a mechanism for recovery from this periodic impact. It is likely that at other times of the year, when exposure is less common, the plants have a chance to replenish depleted energy stores. However the increased incidence of exposure during the study was likely to have reduced this natural resilience. Seagrass meadows under reduced levels of resilience will be in a more vulnerable state to other impacts including those associated with future climate change and anthropogenic disturbance (i.e. poor water quality and/or dredging).

We do not suggest that tidal exposure and solar irradiation are the only factors that influence seagrass condition in Weipa, only that in the timeframe of this study they were likely to be a major contributor to the observed patterns of seagrass change. In other intertidal seagrass meadows subject to large tidal ranges similar effects may also be observed and should be considered. A range of other factors including below-ground biomass and sediment characteristics that were not investigated in this study may also influence long term seagrass dynamics in Weipa and other locations [2], [50].

Other studies have found seasonal declines within an intra-annual cycle in tropical seagrass (above-ground) biomass due to tidal exposure [19], [20], [51], [52]. However, the present study demonstrates that natural multi-year shifting in tidal patterns can explain a longer term inter-annual decline in above-ground biomass. Without detailed analysis of the tidal exposure regime it may not have been obvious what the major driver of seagrass decline was in Weipa, and may have resulted in costly investigations and mitigation to an environmental problem that was linked to natural environmental change. This highlights the importance of developing an appropriate monitoring protocol that includes the interpretation of variable seagrass dynamics in relation to natural environment factors as well as anthropogenic factors.

## References

[1] Waycott M, Duarte CM, Carruthers TJB, Orth RJ, Dennison WC (2009). Accelerating loss of seagrasses across the globe threatens coastal ecosystems.. Proc Natl Acad Sci U S A.

[2] Terrados J, Duarte CM, Fortes MD, Borum J, Agawin NSR (1998). Changes in community structure and biomass of seagrass communities along gradients of siltation in SE Asia.. Est Coast Shelf Sci.

[3] Erftemeijer PLA, Lewis RRR (2006). Environmental impacts of dredging on seagrasses: A review.. Mar Poll Bull.

[4] Orth RJ, Carruthers TJB, Dennison WC, Duarte CM, Fourqurean JW (2006). A global crisis for seagrass ecosystems.. BioScience.

[5] McKenzie LJ (1994). Seasonal changes in biomass and shoot characteristics of a Zostera capricorni Aschers. dominant meadow in Cairns Harbour, northern Queensland.. Aus J Mar Freshwater Res.

[6] Hall MO, Durako MJ, Fourqurean JW, Zieman JC (1999). Decadal changes in seagrass distribution and abundance in Florida Bay.. Estuaries.

[7] Rasheed MA, Unsworth RKF (2011). Long-term climate associated dynamics of a tropical seagrass meadow: implications for the future.. Mar Ecol Prog Ser.

[8] Unsworth RKF, Cullen LC (2010). Recognising the necessity for Indo-Pacific seagrass conservation.. Conserv Lett.

[9] Hemminga MA, Duarte CM (2000).

[10] Campbell SJ, McKenzie LJ, Kerville SP (2006). Photosynthetic responses of seven tropical seagrasses to elevated seawater temperature.. J Exp Mar Biol Ecol.

[11] Collier CJ, Waycott M, Ospina AG (2011). Responses of four Indo-West Pacific seagrass species to shading.. Mar Poll Bull.

[12] Waycott M, Collier C, McMahon K, Ralph PJ, McKenzie LJ, Johnson JE, Marshall PA (2007). Vulnerability of seagrasses in the Great Barrier Reef to climate change - Chapter 8:.. Climate Change and the Great Barrier Reef: A Vulnerability Assessment, Part II: Species and species groups: Great Barrier Reef Marine Park Authority.

[13] Short FT, Neckles HA (1999). The effects of global climate change on seagrasses.. Aquat Bot.

[14] Björk M, Uka J, Weil A, Beer S (1999). Photosynthetic tolerances to desiccation of tropical intertidal seagrasses.. Mar Ecol Prog Ser.

[15] Figueroa FL, Jimenez C, Vinegla B, Perez-Rodriguez E, Aguilera J (2002). Effects of solar UV radiation on photosynthesis of the marine angiosperm *Posidonia oceanica* from Southern Spain.. Mar Ecol Prog Ser.

[16] Trocine RP, Rice JD, Wells GN (1981). Inhibition of seagrass photosynthesis by ultraviolet-B radiation.. Plant Phys.

[17] Pollard PC, Greenway M (1993). Photosynthetic characteristics of seagrasses (*Cymodocea serrulata*, *Thalassia hemprichii* and *Zostera capricorni*) in a low-light environment, with a comparison of leaf-marking and lucunal-gas measurements of productivity.. Aus J Mar Freshwater Res.

[18] Ralph PJ, Burchett MD (1995). Photosynthetic responses of the seagrass *Halophila ovalis* to high irradiance stress, using chlorophyll {Ia} fluorescence.. Aquat Bot.

[19] Erftemeijer PLA, Herman PMJ (1994). Seasonal changes in environmental variables, biomass, production and nutrient contents in two contrasting tropical intertidal seagrass beds in South Sulawesi, Indonesia.. Oecologia.

[20] Stapel J (1997). Biomass loss and nutrient redistribution in an indonesian *Thalassia hemprichii* seagrass bed following seasonal low tide exposure during daylight.. Mar Ecol Prog Ser.

[21] Seddon S, Connolly RM, Edyvane KS (2000). Large-scale seagrass dieback in northern Spencer Gulf, South Australia.. Aquat Bot.

[22] Nienhuis PH, Coosen J, Kiswara W (1989). Community structure and biomass distribution of seagrasses and macrofauna in the Flores Sea, Indonesia.. Neth J Sea Res.

[23] Unsworth RKF, Cullen LC, Pretty JN, Smith DJ, Bell JJ (2010). Economic and subsistence values of the standing stocks of seagrass fisheries: Potential benefits of no-fishing marine protected area management.. Ocean Coast Manage.

[24] Taylor HA, Rasheed MA (2011). Impacts of a fuel oil spill on seagrass meadows in a subtropical port, Gladstone, Australia - The value of long-term marine habitat monitoring in high risk areas.. Mar Poll Bull.

[25] Chartrand KM, Rasheed MA (2009). Port of Weipa Long term seagrass monitoring, 2000–2008..

[26] McKenzie LJ, Finkbeiner MA, Kirkman H, Short FT, Coles RG (2001). Methods for mapping seagrass distribution.. Global Seagrass Research Methods: Elsevier Science B.V., Amsterdam.

[27] Burdick DM, Kendrick GA, Short FT, Coles RG (2001). Standards for seagrass collection, identification and sample design.. Global Seagrass Research Methods Elsevier, Amsterdam.

[28] Mellors JE (1991). An evaluation of a rapid visual technique for estimating seagrass biomass.. Aquat Bot.

[29] Kirkman H (1978). Decline of Seagrass in Northern Areas of Moreton Bay, Queensland.. Aquat Bot.

[30] BOM (2011). Australian Federal Bureau of Meteorology.. http://www.bom.gov.au.

[31] Haapkyla J, Unsworth RKF, Flavell M, Bourne DG, Schaffelke B (2011). Seasonal Rainfall and Runoff Promote Coral Disease on an Inshore Reef.. Plos One.

[32] Carrascal LM, Galvan I, Gordo O (2009). Partial least squares regression as an alternative to current regression methods used in ecology.. Oikos.

[33] Zar JH (1984). Biostatistical Analysis.

[34] Ort DR (2001). When there is too much light.. Plant Phys.

[35] Anthony KRN, Kerswell AP (2007). Coral mortality following extreme low tides and high solar radiation.. Mar Biol.

[36] Collier CJ, Uthicke S, Waycott M (2011). Thermal tolerance of two seagrass species at contrasting light levels: Implications for future distribution in the Great Barrier Reef.. Limnol Ocean.

[37] IPCC (2007). Climate Change 2007: The Physical Science Basis: Summary for Policymakers. Contribution of Working Group I to the Fourth Assessment Report of the Intergovernmental Panel on Climate Change.

[38] Hader DP, Helbling EW, Williamson CE, Worrest RC (2011). Effects of UV radiation on aquatic ecosystems and interactions with climate change.. Photochem Photobiol Sci.

[39] Worrest RC, Hader DP (1989). Effects of stratospheric ozone depletion on marine organisms.. Env.

[40] Coles RG, Lee Long WJ, Watson RA, Derbyshire KJ (1993). Distribution of seagrasses, and their fish and penaeid prawn communities, in Cairns Harbour, a tropical estuary, northern Queensland, Australia.. Aus J Mar Freshwater Res.

[41] Unsworth RKF, De Grave S, Jompa J, Smith DJ, Bell JJ (2007). Faunal relationships with seagrass habitat structure: a case study using shrimp from the Indo-Pacific.. Mar Freshwater Res.

[42] Unsworth RKF, Garrard S, Salinas De Leon P, Sloman KA, Cullen LC (2009). Structuring of Indo-Pacific fish assemblages along the mangrove-seagrass continuum.. Aquat Biol.

[43] McArthur LC, Boland JW (2006). The economic contribution of seagrass to secondary production in South Australia.. Ecol Model.

[44] Kahn AE, Durako MJ (2009). Photosynthetic tolerances to desiccation of the co-occurring seagrasses Halophila johnsonii and Halophila decipiens.. Aquat Bot.

[45] Demmig-Adams B, Adams W, Ebbert V, Logan B, Frank H, Young A, Britton G, Cogdell R (2004). Ecophysiology of the Xanthophyll Cycle.. The Photochemistry of Carotenoids.

[46] Tanaka Y, Nakaoka M (2004). Emergence stress and morphological constraints affect the species distribution and growth of subtropical intertidal seagrasses.. Mar Ecol Prog Ser.

[47] Valentine JF, Duffy JE, Larkum AWD, Orth JJ, Duarte CM (2006). Grazing in seagrass ecosystems.. Seagrasses: Biology, ecology and conservation.

[48] Rasheed MA (2004). Recovery and succession in a multi-species tropical seagrass meadow following experimental disturbance: the role of sexual and asexual reproduction.. J Exp Mar Biol Ecol.

[49] Rasheed MA (1999). Recovery of experimentally created gaps within a tropical Zostera capricorni (Aschers.) seagrass meadow, Queensland Australia.. J Exp Mar Biol Ecol.

[50] Koch MS, Schopmeyer S, Kyhn-Hansen C, Madden CJ (2007). Synergistic effects of high temperature and sulfide on tropical seagrass.. J Exp Mar Biol Ecol.

[51] Brouns JJWM, Heijs FML (1986). Production and biomass of the seagrass *Enhalus acoroides* (L.f.) Royle and its epiphytes.. Aquat Bot.

[52] Brouns JJWM (1987). Aspects of production and biomass of four seagrass species (Cymodoceoideae) from Papua New Guinea.. Aquat Bot.

